# Effects of spaced k-mers on alignment-free genotyping

**DOI:** 10.1093/bioinformatics/btad202

**Published:** 2023-06-30

**Authors:** Hartmut Häntze, Paul Horton

**Affiliations:** Department of Computer Science and Information Engineering, National Cheng Kung University, Tainan City 701, Taiwan; Department of Computer Science and Information Engineering, National Cheng Kung University, Tainan City 701, Taiwan

## Abstract

**Motivation:**

Alignment-free, k-mer based genotyping methods are a fast alternative to alignment-based methods and are particularly well suited for genotyping larger cohorts. The sensitivity of algorithms, that work with k-mers, can be increased by using spaced seeds, however, the application of spaced seeds in k-mer based genotyping methods has not been researched yet.

**Results:**

We add a spaced seeds functionality to the genotyping software PanGenie and use it to calculate genotypes. This significantly improves sensitivity and F-score when genotyping SNPs, indels, and structural variants on reads with low (5×) and high (30×) coverage. Improvements are greater than what could be achieved by just increasing the length of contiguous k-mers. Effect sizes are particularly large for low coverage data. If applications implement effective algorithms for hashing of spaced k-mers, spaced k-mers have the potential to become an useful technique in k-mer based genotyping.

**Availability and implementation:**

The source code of our proposed tool MaskedPanGenie is openly available on https://github.com/hhaentze/MaskedPangenie.

## 1 Introduction

Next-generation sequencing (NGS) produces a vast amount of short amino acid or nucleotide sequences of up to 300 base pairs (bp). These sequences, called reads, do not directly give us an organism’s genome because their position within the genome is unknown. For further analysis, e.g. variant calling, many workflows either assemble reads *de novo* or align them to a reference genome. Aligning full reads by exact matching can be done in linear time, but the presence of variants and sequencing errors requires inexact matching. Dynamic programming algorithms can find optimal solutions for inexact matching with quadratic time complexity (Smith and Waterman 1981; Gotoh 1982). However, faced with the enormous amount of data produced by NGS systems, this quickly became insufficient. Recent methods, such as BWA-MEM (Li 2013), utilize hash functions, advanced indices and seeding techniques to accelerate read alignment further. Nevertheless, they still require a considerable amount of time and computing power.

To further increase genotyping speed and reduce reference bias, alignment-free methods, such as PanGenie (Ebler et al. 2022), were proposed. These methods utilize vast databases of known variants in the human genome, which are available through the sequencing efforts and benchmark standardization of the last decade (e.g. 1000 Genomes Project, Genome in a Bottle). By mapping k-mers of a sample directly to a variant database, genotyping can be sped up immensely. Compared with mapping-based approaches, PanGenie can genotype more than four times faster at 30× coverage and achieves better genotype concordance (Ebler et al. 2022). The acceleration is partially due to the use of exact matching of k-mers, as opposed to inexact matching. However this comes at the cost of not being able to utilize k-mers spanning a read sequence error to identify variants.

Spaced (also know as discontiguous) k-mers provide a possible solution to this problem. This approach was introduced to bioinformatics in the context of homology search (Ma et al. 2002) and was quickly adopted by other applications. To the best of our knowledge, exact matching of spaced k-mers has not yet been applied in alignment-free k-mer based genotyping. This article aims to evaluate the potential spaced k-mers might have on these algorithms. There exist many different algorithms, and evaluating the effect of spaced k-mers on all of them is not feasible. Hence, this study will concentrate on a single application, namely PanGenie (Ebler et al. 2022), which had higher genotype concordance and faster computing time than other applications. The effects of spaced k-mers on this application should give a general impression on possible advantages or problems spaced k-mers introduce to alignment-free genotyping.

## 2 Related work

This section introduces the literature this study is built on. First, it provides an overview of alignment-free genotyping. Second, spaced k-mers are introduced and explained in depth. Lastly, the intersection of both technologies is discussed.

### 2.1 Alignment-free genotyping

Genotyping is the process of determining which genetic variants an individual possesses (Byard and Payne-James 2016). Many genotyping workflows map the reads to a reference genome and then call differences between reads and reference sequence [e.g. BWA-MEM and GATK Haplotype Caller (McKenna et al. 2010; Li 2013)]. However, mapping the reads to the reference genome is computationally expensive and introduces reference bias to the results (Zielezinski et al. 2017). Alignment-free genotyping methods were developed to further accelerate genotyping and reduce reference bias. Their advantage is that they are computationally inexpensive and more robust to shuffling and recombination events, thus being well suited for analyzing populations with high variation in their genome (Vinga 2014). Disadvantages, however, are that symbol order is lost in the majority of alignment-free algorithms (Vinga 2014) and that unknown variants cannot be detected. Two main categories of alignment-free sequence comparisons have been proposed (Vinga and Almeida 2003). The first is based on the frequency of fixed-length k-mers within the reads and the second is based on Kolmogorov complexity and Chaos Theory. This article only considers the first category.

Variants in the genome have specific patterns of k-mers surrounding them. To genotype a known variant, one can count how many and how often surrounding k-mers appear in the reads and calculate likelihoods of reference and alternative alleles. This is much faster than trying to find optimal read alignments, as counting k-mers can be done with almost linear time complexity through exact matching and hash maps.

In recent years, many applications and studies about alignment-free algorithms have been published. Shajii et al. (2016) introduced a lightweight assignment of variant alleles (LAVA). Given a set of SNPs, LAVA constructs a dictionary of mid-size k-mers (*k* = 32), which uniquely belong to these SNPs, as well as a second dictionary of the k-mers in the human genome. Through bipartite matching of k-mers in the reads to the k-mers in the precomputed dictionary up to Hamming distance 1, LAVA can determine if a read belongs to a particular SNP. It then calculates genotypes (homozygous reference, heterozygous, homozygous alternate) with Bayes’ rule using allele frequencies. Denti et al. (2019) extended this approach and introduced a method that can genotype multi-allelic SNPs and indels using exact matching of contiguous k-mers. Similarly, Sibbesen et al. (2018) developed BayesTyper, which exactly matches contiguous k-mers (*k* = 55) to a variant graph and genotypes each variant, including SVs, based on observed k-mer frequencies. BayesTyper then uses an iterative, heuristic algorithm to estimate a subset of most likely haplotypes. They observe that variants that are less than k-1 nucleotides apart will share a k-mer and must thus be clustered together in the variant graph. Additionally, adding a database containing known variation significantly improves the sensitivity of genotyping structural variation. K-mer counts alone are not sufficient to reliable genotype loci that are poorly covered by k-mers, or do not have unique k-mers (Ebler et al. 2022). Ebler et al*.* propose an algorithm, PanGenie, that overcomes this limitation by leveraging linkage disequilibrium (LD) of structures inherent in multiple haplotype-resolved assemblies. LD is the phenomenon that alleles, at population level, are not independent of each other but follow specific patterns. It can be used to make assumptions about genotypes based on the surrounding alleles. For this, PanGenie first builds an acyclic and directed pangenome graph from a callset of multiple haplotype-resolved assemblies. The pangenome graph contains bubbles, i.e. different allele paths, for each variant in the callset. To calculate the likelihood of allele combinations in each bubble, PanGenie compares the expected counts of k-mer unique to the bubble with the actual counts of these k-mer in the reads. Finally, it infers genotypes of poorly covered loci by leveraging LD through a Hidden Markov Model.

This approach is faster and achieves better genotype concordances than most mapping-based algorithms. Improvements are especially notable for large insertions and repetitive regions (Ebler et al. 2022). However, PanGenie only uses contiguous k-mers, even though spaced k-mers have reportedly improved many k-mer-based algorithms (Ma et al. 2002; Leimeister et al. 2014; Wood et al. 2019).

### 2.2 Spaced k-mers

K-mers are substrings of constant length *k*, with *k* being smaller than the length of the reads. Typically, they are contiguous, meaning they capture all consecutive bases within a substring of a nucleotide sequence. Contiguous k-mers have been used in BLAST (Altschul et al. 1990) for local alignment, in de Bruijn-graphs for assembly [e.g. Velvet (Zerbino and Birney 2008)] and in metagenomic sequence classification [e.g. Kraken (Wood and Salzberg 2014)]. Choosing the optimal length *k* is essential. Long k-mers have a high probability of containing sequencing errors and may lose distant homologies. Short k-mers, on the other hand, are not as distinctive and increase the computational burden (Ma et al. 2002).

One method to increase k-mer length, while keeping the risk of missing matches due to sequencing errors low, is approximate k-mer matching (Shajii et al. 2016). In contrast to exact matching, this also matching up to a given Hamming distance. However, while this reduces the effect of sequencing errors, it also decreases the number of identifiable unambiguous loci in the genome (Shajii et al. 2016). For example, allowing a hamming distance of 1, a single k-mer in the reference database could be matched with up to (4)(k) different k-mers in the reads.

Another method that reduces the effect of sequencing errors without affecting the number of identifiable unambiguous loci is exact k-mer matching using spaced seeds. Early applications of spaced seeds can be found in Ma et al. (2002), for faster and more sensitive homology search, as well as by Burkhardt and Kärkkäinen (2003), for faster and/or more efficient filtering than with contiguous k-mers. They can increase the sensitivity of seed-and-extend methods for local alignment (Ma et al. 2002; Gotea et al. 2003; Kiełbasa et al. 2011) and can improve results of alignment-free sequence comparison methods (Leimeister et al. 2014) as well as specificity and sensitivity in metagenomic classification (Břinda et al. 2015; Wood et al. 2019).

Definition 1. A seed *s* is a binary pattern over the symbols 1 and 0, denoting *care* and *don’t care* positions, respectively (Břinda et al. 2015). We use *l* to denote the length of this pattern, and *w* to denote the number of 1’s in the patter, known as the weight of the seed. If the weight and length of *s* are equal, *s* is called contiguous, otherwise spaced. A seed is palindromic if care and do not care positions remain the same, regardless of the direction in which the seed is read.

Definition 2. Given a spaced seed with length *l* and weight w=k, a spaced *k*-mer is a series of *k* characters of a substring of length *l* at care positions of the used seed. For example, applying seed 1101 (two care positions followed by a do not care position and another care position) on the sequence ACTGA, results in the spaced 3-mers ACG and CTA. Two spaced k-mers can be equal, even when the raw sequences differ. For instance, using seed 1101 on the sequences ATGC and ATAC will result in the spaced 3-mer ATC in both cases.

#### 2.2.1 Seed selection

Applications that use contiguous k-mers must choose a suitable weight k to achieve the wanted trade-off between distinctiveness and risk of sequencing errors. For spaced seeds, this gets more complicated. Apart from the weight and length of a seed, care and do not care positions need to be specified, too.

A seed’s ability to detect similar segments between biological sequences is measured in terms of sensitivity, i.e. the probability of generating a hit with a spaced seed in a fixed-length region with a given similarity level. Different seeds have different sensitivity, and selecting a seed with high sensitivity is essential. Ma et al. (2002) argue that the reason for increased sensitivity is that the events of having a match at different positions become more independent for spaced seeds. ‘Generally, the fewer bases shared by a model and its shifted copies, the higher its sensitivity is (Ma et al. 2002).’ The best seeds typically possess little regularity, with consecutive seeds consequently being among the worst (Burkhardt and Kärkkäinen 2003).

Sensitivity can be calculated with dynamic programming (Keich et al. 2004) or a recurrence relation (Choi and Zhang 2004). Unfortunately the problem is NP-hard (Ma and Li 2007), with both the number of possible spaced seeds, and the time to calculate the hit probability, i.e. sensitivity, of a given spaced seed increasing exponentially in l−w. Thus in general spaced seeds can only be optimized approximately.

As a heuristic, Ilie and Ilie (2007) introduce a new measure, overlap complexity, and show that it is experimentally well correlated to sensitivity. They give an algorithm that starts with a randomly selected initial seed and tries to improve it iteratively. Ilie et al. (2011) implement this algorithm in their software SpEED which executes the algorithm multiple times for different seed lengths. Through these approximations, SpEED can compute highly sensitive multiple spaced seeds in a few seconds.

### 2.3 Spaced k-mers in alignment-free genotyping

As demonstrated by Ma et al. (2002) and others, spaced seeds are effective seed-and-extend local alignment applications such as homology search and read mapping. However those findings cannot be directly transferred to alignment-free genotyping; where the measure of success is not the percent accuracy of mapping individual reads, but rather the aggregate effect of the ensemble of read k-mers matched to known variant alleles.

Sequencing errors and mutations affect the proportion of k-mers that can be mapped to different variant alleles at a locus. Thus theoretically spaced k-mers might be able to increase this proportion in favour of the correct allele. Whether this is true in practice is however not self-evident; especially when sequence errors are infrequent. For example, reads in this study were sequenced with an Ilumina HiSeq X Ten platform with a median error rate of only 0.087% (Stoler and Nekrutenko 2021) per base.

This study aims to investigate the practical advantages (if any) of using spaced k-mers for one genotyping method, namely PanGenie (Ebler et al. 2022).

## 3 Methodology

### 3.1 Problem formalization

Given a set of *reads*, a reference genome *ref* and a haplotype resolved file of known variants *vcf*, PanGenie (Ebler et al. 2022) can be used to predict haplotypes $h_{C}$ by using a contiguous seed *C*. To analyse spaced seeds’ effects on genotyping, this study proposes an adapted software *MaskedPanGenie*, which extends PanGenie with a spaced seed functionality. Given a space seed *S*, MaskedPanGenie can predict haplotypes $h_{S}$ by using spaced-k-mers in reads, instead of contiguous k-mers. It is written in C++ and publicly available at https://github.com/hhaentze/MaskedPangenie. MaskedPanGenie requires the occurrences of spaced k-mers in the reads as input. We count spaced k-mers by combining the counting tool Jellyfish (Marçais and Kingsford 2011) with our own helper application *MaskJelly* (https://github.com/hhaentze/MaskJelly). We aim to investigate the effects of do not care positions on genotyping by comparing the results of genotyping with seeds *C* and *S* (of equal weight). However, apart from having do not care positions, spaced seed *S* is also longer than the contiguous seed *C*. Hence, differences in the genotyping results with *C* and *S* could also be due to the different lengths. Thus, we include a contiguous seed $C^{\prime }$ with length equal to *S* as a second control group. If do not care positions do not significantly affect the genotyping process, similar results can be expected for spaced seed *S* and contiguous seeds of equal weight (*C*) or length ($C^{\prime }$).


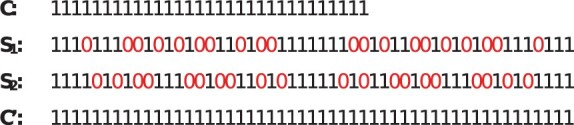

1. **C** (control group 1) A mid-sized contiguous seed with weight and length of 31. (default k-mer size in PanGenie)
2. **S_1_ and S_2_** Spaced seeds with weight of 31 and length of 51. Length and patterns of do not care positions are calculated with the help of SpEED (Ilie et al. 2011). We chose the first and second most sensitive patterns according to SpeEED.
3. **C’** (control group 2) A long-sized contiguous seed with weight and length of 51.

We use MaskedPanGenie to calculate callsets for each seed and asked which callset $h_{C}$, $h_{S_{1,2}}$, or $h_{C^{\prime }}$ is closest to a given ground truth.


$$\begin{array}{c} h_{C}(\text{reads})=\mathrm{MaskedPanGenie}(\text{reads},\text{ref},\text{vcf}|C)\\ h_{S_{x}}(\text{reads})=\mathrm{MaskedPanGenie}(\text{reads},\text{ref},\text{vcf}|S_{1,2})\\ h_{C^{\prime }}(\text{reads})=\mathrm{MaskedPanGenie}(\text{reads},\text{ref},\text{vcf}|C^{\prime }) \end{array}$$

Variants in the predicted callsets can be either absent (0|0), ie. both alleles are in line with the reference genome, heterozygous (1|0), homozygous (1|1) or undefined (*|*). The differences between the predicted sets and the true set of haplotypes are measured in terms of recall, precision and F-score.


$$\begin{array}{c} \begin{array}{c} \text{Recall}=\frac{\text{TP}}{\text{TP}+\text{FN}}\;\;\;\text{Precision}=\frac{\text{TP}}{\text{TP}+\text{FP}} \end{array}\\ F-\text{score}=2\times \frac{\text{precision}\times \text{recall}}{\text{precision}+\text{recall}} \end{array}$$

This study defines true positive (TP), false positive (FP) and false negative (FN) according to the most stringent model of the ‘Best practices for benchmarking germline small-variant calls in human genomes’ (Krusche et al. 2019). Only sides with identical alleles and genotype in truth and callset are counted as TPs. Another metric that can be used for comparing call sets is genotype concordance, i.e. the proportion of correctly genotyped variants. However, Ebler et al. (2022) argue that genotype concordance is flawed if callsets contain variants originating from multiple samples because the number of absent variants (0|0) will be higher than the number of variants with true genotypes 0|1 or 1|1. To adjust for unequal numbers of 0|0, 0|1 and 1|1, they use a weighted genotype concordance (wGC), which is computed as: where $T_{x|y}$ and $F_{x|y}$ denote the number of correctly and erroneously classified variants of genotype x|y respectively.


$$\begin{array}{c} \begin{array}{c} \text{conc}(x|y)=\frac{T_{x|y}}{T_{x|y}+F_{x|y}} \end{array}\\ \text{wGC}=\frac{\text{conc}(0|0)+\text{conc}(0|1)+\text{conc}(1|1)}{3} \end{array}$$

This study reports adjusted versions of those metrics, which only include variants that are part of the given known variants file. Variants, which are undefined in the ground-truth set, are removed from both sets. Following the evaluation procedure of PanGenie (Ebler et al. 2022); we use a kind of leave-one-out cross validation in which when calling variants on an individual, the pan-genome used is constructed based the rest of the individuals. To investigate whether sequencing depth has an impact on genotyping with spaced k-mers, we test MaskedPangenie on both low (5×) and high (30×) coverage data. We evaluate genotyping separately for SNVs, indels and SVs. The tool we wrote for the calculation can be accessed at https://github.com/hhaentze/GenotypeConcordance.

### 3.2 Choosing a spaced seed

#### 3.2.1 Seed structure

For the weight of the spaced seed *S* we choose the k-mer length used in the original PanGenie study, which is 31. The length of *S* needs to be above 31 and preferably lower than 76, which is half the length of the reads used in this experiment. Seed lengths ≥76 would reduce the maximum number of unique k-mers (e.g. 31 for a weight of 31) that can cover a single nucleotide variation. The first and last positions of the seeds are always ‘1’. PanGenie uses canonical representations of k-mers. Canonical means that k-mers are considered equivalent to their reversed compliment, and the lexicographically smaller version serves as a representative. To guarantee that spaced k-mer from forward and reverse strand have the same canonical form, we require *S* to be palindromic. That is, care and do not care positions must be arranged symmetrically.

#### 3.2.2 Number of possible spaced seeds

Given a weight of *w* and a length of *l* there are generally $\left(\begin{array}{c} l\\ w \end{array}\right)$ different seeds. Previously introduced constraints lower this number. Each seed should start and end with ‘1’ so the number of possible seeds |*S*| can be reduced to $\left(\begin{array}{c} l-2\\ w-2 \end{array}\right)$. The palindromic constraint further reduces this to:


$$|S|=\left(\begin{array}{c} \left\lfloor \frac{l}{2}\right\rfloor -1\\ \left\lfloor \frac{w}{2}\right\rfloor -1 \end{array}\right)$$

For instance, given a weight of 31, there are $\left(\begin{array}{c} 19\\ 14 \end{array}\right)=11\;628$, and $\left(\begin{array}{c} 24\\ 14 \end{array}\right)=1\;961\;256$ possible seeds for lengths 41 and 51 respectively.

#### 3.2.3 Application of SpEED

To obtain a good palindromic spaced seed *S* with weight 31, we use the tool SpEED (Ilie et al. 2011) to create a spaced seed with length 16 and extend the obtained seed with its mirrored version on its right side to ensure that it is palindromic. For the expected similarity level between homologous regions, we choose 95%, as recommended in the SpEED documentation. For the length of homologous regions, we somewhat arbitrarily choose 76, which is half the read length.


SpEED 16 1 0.95 76

The most sensitive seed obtained after 4680 iterations was 11101110010101001101001111, with a sensitivity of 0.999, given the specified parameters. Mirroring and extending the seed results in the seed $S_{1}$, with a length of 51. To construct $S_{2}$ we proceeded in the same way, but instead used the second most sensitive seed (also with a sensitivity of ≈ 0.999) obtained through SpEED.


$$\begin{array}{c} S_{1}=111011100101010011010011111110010110010101001110111\\ S_{2}=111101010011100100110101111101011001001110010101111 \end{array}$$

Note that Ilie et al. (2011) tested their algorithm for multiple spaced seeds up to a weight of 18. PanGenie, on the other hand, is designed for mid-size k-mers with a weight of 31. It is unclear if SpEED can find a good single seed for this weight. However, the complexity of the problem makes it hard to find more optimal solutions.

### 3.3 Statistics

To determine whether the null hypothesis that both methods are equally good can be rejected, we conducted two-sided significance tests. We adopted the Wilcoxon signed-rank test (Wilcoxon 1992) with exactly calculated *P*-values as a testing procedure suitable for paired data with small sample size (Altman et al. 1983; Mundry and Fischer 1998). For a sample size n=9 the smallest possible *P*-value is $(2)(2^{-9})\approx 0.004$.

#### 3.3.1 Consideration of multiple testing

This study tests significance with regard to three variant types {SNV, indel, SV}, two read-coverages {5×, 30×}, and four metrics (precision, recall, F1-Score, wGC), resulting in a total of m=(3)(2)(4)=24 different tests. Therefore we considered if it would be appropriate to apply a standard method for multiple testing correction. The well known Bonferroni provides a strict upper bound on a corrected *P*-value by simply multiplying by *m*. With a sample size of 9, the lowest possible corrected *P*-value would be (24)(0.004)=0.096>5%, excluding any possibility of obtaining statistically significantly results. Less stringent tests such as by Westfall/Young or Benjamin/Hochberg are also problematic because they require ranking individual *P*-values, but small sample size signed-rank tests tend to produce many individual *P*-values of equal value.

Faced with these difficulties, we decided to report individual *P*-values without multiple testing correction. The key caveat is that when interpreting this work the results of all of the tests should be considered together, rather than focusing on a few tests with particularly low *P*-values.

## 4 Data

### 4.1 Pangenome reference

We constructed a haplotype-resolved pangenome graph for the same eleven unrelated individuals as in the study by Ebler et al. (2022) (NA19238, NA19239, HG00731, HG00732, HG00512, HG00513, NA12878, HG02818, HG03125, NA24385, HG03486). For this, we used the merged variation calls, as described by Ebert et al. (2021) and Chaisson et al. (2019), and filtered for variants shared by at least one of the eleven samples, where the alternative genotype of at least 80% of the samples is known. The number of variants covered by the pangenome graph is reported in Table 1.

**Table 1.** Number of distinct variant alleles covered by pangenome graph.

SNV	Indel (<50 bp)	SV (≥50 bp)
11 385 979	2 006 331	69 803

For the leave-one-out cross-validation, we use subsets of the pangenome, which do not include the haplotypes of the tested individual, respectively. The excluded haplotypes are regarded as ground truth. Following Ebler et al. (2022), this study deletes variants not covered by the tested individual’s true haplotype or the pangenome’s remaining haplotypes.

### 4.2 Reads

The test data consists of sequencing reads of 9 out of the 11 individuals used to build the pangenome reference [NCBI Sequence Read Archive (Leinonen et al. 2011), run ids: SRR7782675, SRR7782676, SRR7782672, SRR7782673, SRR7782690, SRR7782691, SRR7782683, SRR7782682, and SRR7782670]. All reads were sequenced within the same BioProject (accession number PRJNA477862) by an Illumina HiSeq X Ten system using a WGS strategy, a coverage of 30× and paired layout. The two remaining individuals (HG03125 and HG03486) are not part of the BioProject, so we did not include them in this study’s workflow to avoid possible noise introduced by different sequencing platforms.

We sampled the reads down to a target coverage of 5× to evaluate the effects of spaced seeds on reads with lower coverage. For this, we aligned the reads to the reference genome with BWA-MEM (Li 2013) and then used Picard DownsampleSam (Broad Institute 2022) with a fraction size of one-sixth. DownsampleSam also includes reads that could not be aligned to the reference genome, which are randomly sampled down to the same fraction.

## 5 Results

### 5.1 Effects on genotyping of SNV, indel, and SV

We genotyped reads with 5× and 30× coverage from nine samples using MaskedPanGenie with four different seeds; two spaced seed $S_{1}$ and $S_{2}$ as well as their contiguous counterparts *C* and $C^{\prime }$. Tables 2 and 3 list the resulting performances for each type of variant. *P*-values and test statistics are listed in the supplementary material.

**Table 2.** Genotyping results for *5-foldcoverage*.

Variant	Seed	wGC	Precision	Recall	F-score
SNV	C	0.877 (SE = 0.005)	0.896 (SE = 0.005)	0.842 (SE = 0.006)	0.868 (SE = 0.005)
	C’	0.877 (SE = 0.007)	0.855 (SE = 0.010)	0.828 (SE = 0.008)	0.841 (SE = 0.009)
	$S_{1}$	**0.905** (SE = 0.004)	**0.896** (SE = 0.004)	**0.869** (SE = 0.005)	**0.883** (SE = 0.005)
	$S_{2}$	0.902 (SE = 0.004)	0.892 (SE = 0.004)	0.866 (SE = 0.005)	0.879 (SE = 0.004)
Indel	C	0.774 (SE = 0.003)	**0.817** (SE = 0.002)	0.722 (SE = 0.004)	0.767 (SE = 0.003)
	C’	0.800 (SE = 0.004)	0.772 (SE = 0.004)	0.729 (SE = 0.005)	0.750 (SE = 0.005)
	$S_{1}$	**0.825** (SE = 0.003)	0.793 (SE = 0.002)	**0.766** (SE = 0.004)	**0.779** (SE = 0.003)
	$S_{2}$	0.822 (SE = 0.003)	0.789 (SE = 0.003)	0.762 (SE = 0.004)	0.776 (SE = 0.003)
SV	C	0.650 (SE = 0.003)	**0.613** (SE = 0.003)	0.579 (SE = 0.004)	0.596 (SE = 0.003)
	C’	0.641 (SE = 0.004)	0.569 (SE = 0.006)	0.551 (SE = 0.005)	0.560 (SE = 0.006)
	$S_{1}$	0.675 (SE = 0.003)	0.599 (SE = 0.004)	**0.597** (SE = 0.003)	0.598 (SE = 0.003)
	$S_{2}$	**0.676** (SE = 0.003)	0.602 (SE = 0.003)	0.596 (SE = 0.003)	**0.599** (SE = 0.003)

The highest results for each metric and variant are written in bold.

**Table 3.** Genotyping results for *30-fold coverage*.^a^

Variant	Seed	wGC	Precision	Recall	F-score
SNV	C	0.942 (SE = 0.001)	0.977 (SE = 0.001)	0.937 (SE = 0.001)	0.957 (SE = 0.001)
	C’	**0.972** (SE = 0.001)	0.978 (SE = 0.001)	0.964 (SE = 0.001)	0.971 (SE = 0.001)
	$S_{1}$	0.972 (SE = 0.001)	**0.980** (SE = 0.001)	**0.966** (SE = 0.001)	**0.973** (SE = 0.001)
	$S_{2}$	0.969 (SE = 0.001)	0.975 (SE = 0.001)	0.963 (SE = 0.002)	0.969 (SE = 0.001)
Indel	C	0.825 (SE = 0.005)	**0.889** (SE = 0.004)	0.790 (SE = 0.007)	0.836 (SE = 0.006)
	C’	0.884 (SE = 0.006)	0.885 (SE = 0.004)	0.836 (SE = 0.011)	0.859 (SE = 0.007)
	$S_{1}$	**0.891** (SE = 0.006)	0.887 (SE = 0.004)	**0.849** (SE = 0.010)	**0.867** (SE = 0.007)
	$S_{2}$	0.888 (SE = 0.006)	0.880 (SE = 0.004)	0.846 (SE = 0.010)	0.862 (SE = 0.007)
SV	C	0.682 (SE = 0.004)	**0.671** (SE = 0.004)	0.616 (SE = 0.005)	0.642 (SE = 0.004)
	C’	0.701 (SE = 0.005)	0.660 (SE = 0.004)	0.618 (SE = 0.007)	0.638 (SE = 0.005)
	$S_{1}$	**0.719** (SE = 0.005)	0.670 (SE = 0.004)	**0.645** (SE = 0.006)	**0.657** (SE = 0.004)
	$S_{2}$	0.716 (SE = 0.005)	0.664 (SE = 0.004)	0.642 (SE = 0.006)	0.653 (SE = 0.005)

a Metrics and standard error of the mean (SE) for each group. Results are averaged over all nine samples and separated by variant and seed type. C, mid-sized contiguous seed; C’, long-sized contiguous seed; S, spaced seed. The highest results for each metric and variant are written in bold.

Recall of genotyping with both spaced seeds $S_{1}$ and $S_{2}$ is significantly higher for all variant groups. This can be observed for all samples at both 30× and 5× coverage. Effects on precision are more diverse. At 5× coverage, precision of $S_{1}$ and $S_{2}$ is significantly higher than that of long-size seed $C^{\prime }$, but worse than that of mid-size seed *C*. However, at 30× coverage, effects of $S_{1}$ and $S_{2}$ are contrary. For instance, $S_{1}$ increases precision of genotyping SNV, compared to both contiguous seeds, while $S_{2}$ decreases precision.

**Figure 1.**
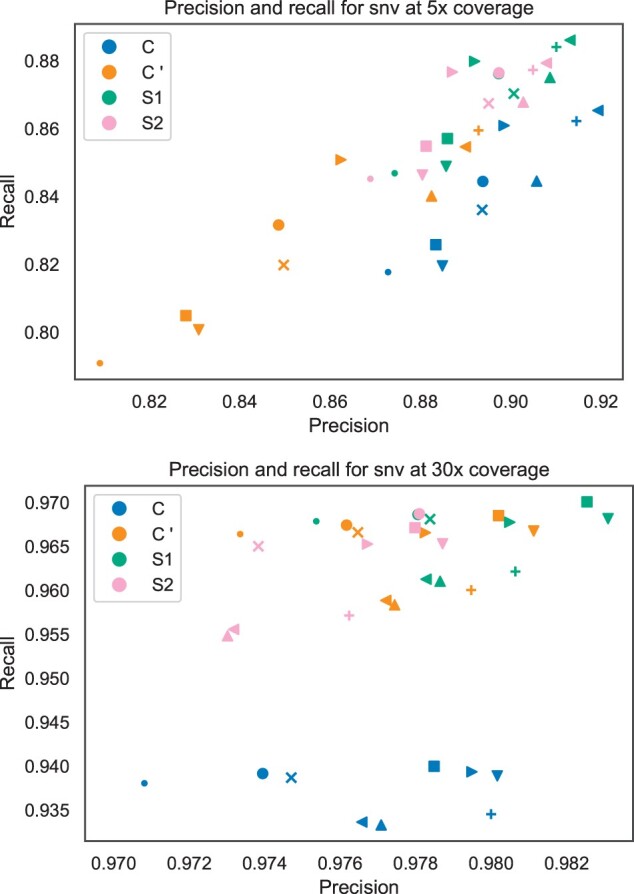
Scatter plot of precision and recall of genotyping SNV with different seeds at 5× and 30× read coverage. Color indicates the type of seed used. Each sample is marked through a unique shape. NA19238:△, NA19239: ⊲, HG00731: □, HG00732: ×, HG00512: ◯, HG00513: ○, NA12878: ▽, HG02818: +, NA24385: ▹.

**Figure 2.**
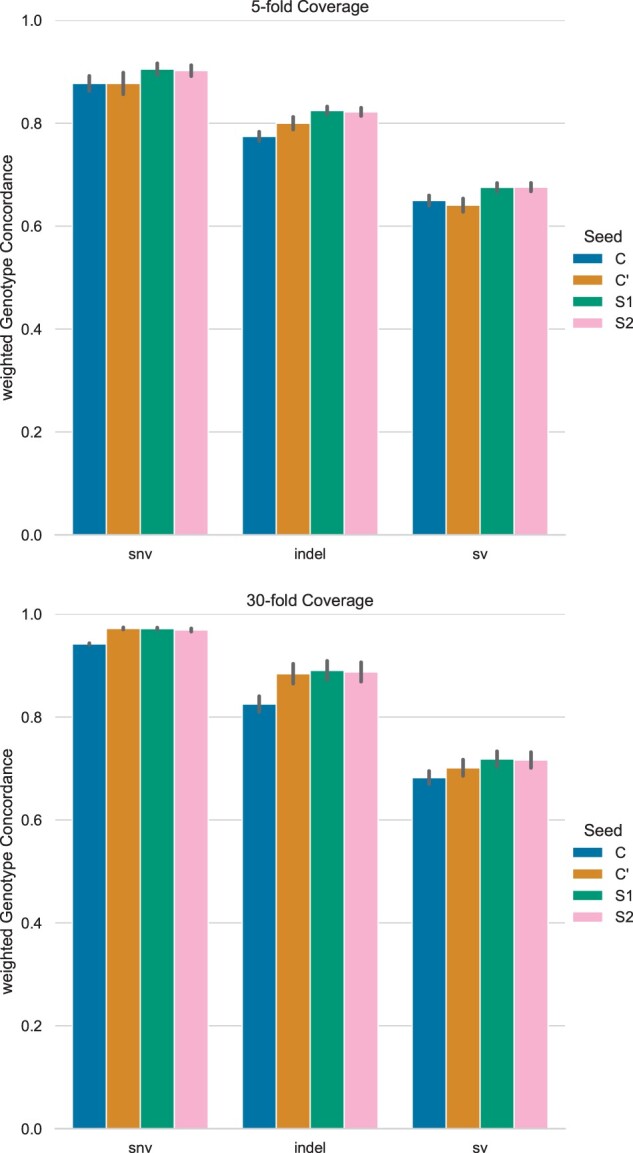
Average weighted genotype concordance and standard deviation of genotyping with different seeds at 5× and 30× coverage.

Nevertheless, the increases in recall are larger than the decreases in precision (Fig. 1). Hence, F1-scores and wGC of $S_{1,2}$ are significantly larger in almost all test cases, as seen in Fig. 2. Compared to the mid-size seed *C*, $S_{1,2}$ show considerable improvements, regardless of coverage. The highest difference can be noted for indel, with an average increase in wGC of 6.5% at 30× coverage. Compared to the long-size seed $C^{\prime }$, F1-score and wGC of $S_{1,2}$ at 5× coverage are significantly larger, too. However, at 30× coverage $S_{2}$ performs worse than $C^{\prime }$ and the effect sizes of other metrics are smaller than at 5× coverage. In fact, improvements of both $S_{1,2}$ and $C^{\prime }$, compared to *C*, correlate positively.


(1) $$\begin{array}{c} {\text{differences}}_{S_{1}}=\mathrm{results}(S_{1})-\mathrm{results}(C)\\ {\text{differences}}_{C^{\prime }}=\mathrm{results}(C^{\prime })-\mathrm{results}(C)\\ r_{1}=\mathrm{Cor}({\text{differences}}_{S_{1}},{\text{differences}}_{C^{\prime }})=0.92\\ r_{2}=\mathrm{Cor}({\text{differences}}_{S_{2}},{\text{differences}}_{C^{\prime }})=0.91 \end{array}$$

The correlation suggests that the length of spaced seeds, although not the only reason, does contribute to their performance. Nevertheless, for more complex variants, i.e. indel and SV, spaced seeds $S_{1,2}$ perform notably better than the long contiguous seed $C^{\prime }$.

### 5.2 Genotype sample NA12878

This section analyzes the effects of genotyping using $S_{1}$ on the arbitrarily selected sample NA12878, at 30× coverage. A breakdown over the total number of genotyped alleles is listed in Table 4. The pangenome graph used for genotyping consists of almost 13 million variant alleles (variants at multiallelic sites are counted separately). MaskedPanGenie correctly genotyped 93% of those alleles when used with seed *C*. For both long-sized seed $C^{\prime }$ and spaced seed $S_{1}$, 97% of alleles could be correctly genotyped. The sets of the 7% and 3% of alleles that were incorrectly genotyped intersect. That is, 342 361 alleles could not be correctly genotyped, regardless of the seed used (40% of incorrect genotyped alleles for seed *C* and 86% for seed $S_{1}$). Regarding the set difference, in absolute numbers, using spaced seed $S_{1}$ resulted in 465 223 more correctly genotyped alleles than for contiguous seed *C*. Many of those alleles were classified as ‘undefined’ when genotyped with *C*. MaskedPanGenie could not calculate a corresponding genotype at all because variant alleles were not covered by any unique k-mers. This is especially pronounced for genotyping of indels. Using seed *C* resulted in 157 257 undefined alleles. For spaced seed $S_{1}$ and long-sized seed $C^{\prime }$, however, only 12 813 and 10 454 alleles were classified as undefined, respectively, 93% less. The decreased number of undefined alleles indicates that MaskedPanGenie could assign unique k-mers to more variant alleles for both $S_{1}$ and $C^{\prime }$.

**Table 4.** Number of correctly and incorrectly genotyped variant alleles for sample NA12878 at 30× coverage.

Variant	Seed	Total alleles	Correct	Incorrect	Proportion
SNV	C	10 963 516	10 384 838	578 678	0.95
	C’	10 963 516	10 739 551	223 965	0.98
	$S_{1}$	10 963 516	10 720 135	243 381	0.98
Indel	C	1 935 511	1 664 769	270 742	0.86
	C’	1 935 511	1 786 510	149 001	0.92
	$S_{1}$	1 935 511	1 792 129	143 382	0.93
SV	C	60 348	45 575	14 773	0.76
	C’	60 348	47 749	12 599	0.79
	$S_{1}$	60 348	48 141	12 207	0.80
Total	C	12 959 375	12 095 182	864 193	0.93
	C’	12 959 375	12 573 810	385 565	0.97
	$S_{1}$	12 959 375	12 560 405	398 970	0.97

### 5.3 Runtime

Tests were executed on a Linux server with an AMD Ryzen 9 5950X 16-Core Processor and 125 GB of RAM. We used two threads for k-mer counting and 16 for the core genotyping algorithms.

Times for genotyping sample NA12878 with 30× coverage are reported in Table 5. PanGenie’s workflow can be broken down into 1. reading of input files, 2. counting of k-mers, 3. identifying unique k-mers and 4. running the HMM. MaskedPanGenie skips the second step, as k-mer counting is done in a preprocessing step. This could result in a faster execution time of MaskedPanGenie (excluding preprocessing), which the version in this study yet fails to achieve, due to parallelization issues. The additional preprocessing step, required for counting spaced k-mers, increases execution time of MaskedPanGenie significantly. PanGenie is more than twice as fast as its masked counterpart. Note that differences in runtime are primarily due to a simplified implementation and not because of theoretical limitations. One reason for the delay is the naive implementation of MaskJelly, which is only intended as a prototype helper tool and effectively uses only one thread, at the moment. We expect that using a suitable hashing algorithm and integrating the counting of spaced k-mers directly into MaskedPanGenie, similar to the counting of contiguous k-mers in PanGenie, can greatly reduce runtime.

**Table 5.** Runtime of PanGenie and MaskedPanGenie for genotyping sample NA12878 with 30× coverage.^a^

Workflow	Preprocessing	Execution	Total
PanGenie (*k* = 31)	–	3 h:07 min	3 h:07 min
PanGenie (*k* = 51)	–	3 h:12 min	3 h:12 min
MaskedPanGenie	4 h:24 min	3 h:27 min	7 h:51 min

a **Execution**: 1. reading of input files, 2. counting of k-mers (only PanGenie), 3. identifying unique k-mers and 4. running the HMM. **Preprocessing**: MaskedPanGenie, additionally, requires one preprocessing step, in which spaced seeds are counted with the help of MaskJelly.

## 6 Discussion

Spaced seeds can increase recall in homology search (Ma et al. 2002) metagenomic classification (Wood et al. 2019) and phylogenetic reconstruction (Leimeister et al. 2014). Motivated by this, we analysed the effects of spaced seeds on alignment-free genotyping with PanGenie. We found that the spaced seed we tested can increase recall, F1-score, and weighted genotype concordance. One reason for this is likely the increased length of a spaced seed. In fact, at 30× coverage, spaced, and long contiguous seeds have a similar impact on genotyping of SNPs with MaskedPanGenie. Furthermore, for both spaced seed and long-sized contiguous seed, MaskedPanGenie can assign unique k-mers to more variant alleles than for a mid-size contiguous seed. Even so, more than seed length is needed to fully explain the improved performance observed with the spaced seed. Compared to contiguous long-sized seeds, the spaced seeds performed significantly better in all metrics at 5× coverage and significantly improved weighted genotype concordance and F1-score of genotyping indel and SV at 30× coverage. Presumably this is due to the do not care positions giving the spaced seed robustness to sequencing errors.

Comparing the two contiguous seeds, this study reported higher recall for a long contiguous seed, than for a mid-sized seed. This is the opposite of conventional expectations for contiguous seeds: Longer k-mers are more likely to contain sequencing errors and, thus, should be less sensitive for homology search. This seeming contradiction can be explained by the differences between the algorithms used for homology search and genotyping. In genotyping, a reduced seed sensitivity affects all alleles at a loci. A reduction in k-mer hits to an alternative allele may be balanced out by a similar reduction in k-mer hits to the reference allele, so the allele probabilities remain the same. Additionally, for large k, more k-mers can be uniquely assigned to a variant, compared to small k. This makes it easier to predict haplotype paths correctly, increasing recall. That said, the effect should be even more dominant for precision. We cannot explain why this is not observable in the data.

Another reason for the increased performance of the longer seed in our experiments might be the high quality of the reads. Other platforms intended for sequencing small genomes, such as Illumina MiniSeq, have up to seven times higher error rates compared to Illumina HiSeq X Ten (Stoler and Nekrutenko 2021). Long k-mers will likely perform worse for reads from these systems. Spaced seeds, on the other hand, might show even larger improvements.

A problem when using spaced k-mers is the increased runtime for k-mer hashing. MaskedPanGenie is much slower than PanGenie. However, this is mainly due to the simplified implementation of MaskedPanGenie. Algorithms that accelerate this process exist (Girotto et al. 2018; Petrucci et al. 2020), but they are not yet implemented in sophisticated software solutions. For further research, it could be of use to develop a spaced k-mer counting tool similar to Jellyfish (Marçais and Kingsford 2011). Implementing such a tool could reduce runtime considerably. Finally, we suspect that spaced k-mers are able to increase performance of other alignment-free genotyping tools, too, as the underlying mechanics of analyzing k-mer frequencies are similar between most applications. For future research it would be interesting to validate this hypothesis by adding spaced k-mer support to other alignment-free genotyping tools.

## 7 Conclusion

This study proposes a new software MaskedPanGenie, which extends the alignment-free k-mer-based genotyper PanGenie with a spaced seed functionality. Haplotypes are calculated for nine samples using contiguous and spaced seeds, respectively. Applying a spaced seed significantly improves recall, weighted genotype concordance, and F1-score for genotyping with low coverage. These improvements are greater than what could be achieved by increasing the length of contiguous k-mers alone. Effects on precision are mixed and hard to evaluate. Running time is increased but in principle that could be ameliorated by incorporating known special techniques for fast spaced seed hash computation.

We conclude that spaced seeds may become a valuable technique for improving alignment-free genotyping, particularly for low read-coverages.

## Supplementary Material

btad202_Supplementary_Data

## Data Availability

The data underlying this article are available in the article and in its online supplementary material.
