# Supplementary material for: Effects of spaced k-mers on alignment-free genotyping
Source: Bioinformatics. 2023 Jun 30;39(Suppl 1):i213–21. doi: 10.1093/bioinformatics/btad202 (PMC10311327; doi:10.1093/bioinformatics/btad202)
Supplement: btad202_Supplementary_Data [file btad202_supplementary_data.pdf]

Effects of Spaced k-mers on Alignment-Free Genotyping: supplemental document

1. SIGNIFICANCE TESTS

			wGC	Precision	Recall	F-score
Variant	Con. Seed	Sp. Seed				
snv	C	S_1	0.004 (W=0)	0.910 (W=21)	0.004 (W=0)	0.004 (W=0)
		S_2	0.004 (W=0)	0.039 (W=-5)	0.004 (W=0)	0.004 (W=0)
	C′	S_1	0.004 (W=0)	0.004 (W=0)	0.004 (W=0)	0.004 (W=0)
		S_2	0.004 (W=0)	0.004 (W=0)	0.004 (W=0)	0.004 (W=0)
indel	C	S_1	0.004 (W=0)	0.004 (W=-0)	0.004 (W=0)	0.004 (W=0)
		S_2	0.004 (W=0)	0.004 (W=-0)	0.004 (W=0)	0.004 (W=0)
	C′	S_1	0.004 (W=0)	0.004 (W=0)	0.004 (W=0)	0.004 (W=0)
		S_2	0.004 (W=0)	0.004 (W=0)	0.004 (W=0)	0.004 (W=0)
sv	C	S_1	0.004 (W=0)	0.004 (W=-0)	0.004 (W=0)	0.129 (W=9)
		S_2	0.004 (W=0)	0.004 (W=-0)	0.004 (W=0)	0.004 (W=0)
	C′	S_1	0.004 (W=0)	0.004 (W=0)	0.004 (W=0)	0.004 (W=0)
		S_2	0.004 (W=0)	0.004 (W=0)	0.004 (W=0)	0.004 (W=0)

Table S1. **5x coverage:** P-values and test statistic W for differences between results of spaced seeds S_1 and S_2 compared to contiguous seeds C and C′. Statistics were calculated with the two-sided exact Wilcoxon signed-rank test. W is the sum of the ranks of the differences above or below zero, whichever is smaller. Positive W indicate a higher group average for the spaced seed execution and negative W a higher group average of the contiguous seed execution.

			wGC	Precision	Recall	F-score
Variant	Con. Seed	Sp. Seed				
snv	C	S_1	0.004 (W=0)	0.004 (W=0)	0.004 (W=0)	0.004 (W=0)
		S_2	0.004 (W=0)	0.129 (W=-9)	0.004 (W=0)	0.004 (W=0)
	C′	S_1	0.004 (W=-0)	0.004 (W=0)	0.004 (W=0)	0.004 (W=0)
		S_2	0.004 (W=-0)	0.012 (W=-2)	0.008 (W=-1)	0.012 (W=-2)
indel	C	S_1	0.004 (W=0)	0.203 (W=-11)	0.004 (W=0)	0.004 (W=0)
		S_2	0.004 (W=0)	0.004 (W=-0)	0.004 (W=0)	0.004 (W=0)
	C′	S_1	0.004 (W=0)	0.004 (W=0)	0.004 (W=0)	0.004 (W=0)
		S_2	0.004 (W=0)	0.008 (W=-1)	0.004 (W=0)	0.008 (W=1)
sv	C	S_1	0.004 (W=0)	0.098 (W=-8)	0.004 (W=0)	0.004 (W=0)
		S_2	0.004 (W=0)	0.004 (W=-0)	0.004 (W=0)	0.004 (W=0)
	C′	S_1	0.004 (W=0)	0.004 (W=0)	0.004 (W=0)	0.004 (W=0)
		S_2	0.004 (W=0)	0.074 (W=7)	0.004 (W=0)	0.004 (W=0)

Table S2. 30x coverage: P-values and test statistic W for differences between results of spaced seeds S_1 and S_2 compared to contiguous seeds C and C′. Statistics were calculated with the two-sided exact Wilcoxon signed-rank test. W is the sum of the ranks of the differences above or below zero, whichever is smaller. Positive W indicate a higher group average for the spaced seed execution and negative W a higher group average of the contiguous seed execution.
